# Intracranial hemorrhage after ischemic stroke in patients on direct oral anticoagulants: results from a prospective observational study

**DOI:** 10.1186/s42466-026-00462-y

**Published:** 2026-02-26

**Authors:** Jan C. Purrucker, Kirsten Haas, Marilen Sieber, Viktoria Rücker, Uwe Malzahn, Karl Georg Haeusler, Christian H. Nolte, Maria Magdalena Gabriel, Johannes Schiefer, Sven Poli, Dominik Michalski, Hassan Soda, Georg Royl, Johannes Meyne, Darius G. Nabavi, Pascal Mosimann, Adrian Heeger, David Kinzler, Patrick Müller, Pascal Rappard, Timolaos Rizos, Peter U. Heuschmann, Roland Veltkamp

**Affiliations:** 1 Department of Neurology, Heidelberg University Hospital, Heidelberg, Germany; 2 Institute of Clinical Epidemiology and Biometry, Julius-Maximilians-Universität Würzburg (JMU), Würzburg, Germany; 3 Clinical Trial Center, University Hospital Würzburg, Würzburg, Germany; 4 Department of Neurology, Universitätsklinikum Ulm, Ulm, Germany; 5 Center for Stroke Research Berlin (CSB), Berlin, Germany; 6 Department of Neurology With Experimental Neurology, Berlin Institute of Health (BIH), Charité Universitätsmedizin Berlin, Berlin, Germany; 7 Department of Neurology, Hannover Medical School, Hannover, Germany; 8 Department of Neurology, University Hospital RWTH Aachen, Aachen, Germany; 9 Department of Neurology & Stroke, Tübingen University Hospital, Tübingen, Germany; 10 Hertie Institute for Clinical Brain Research, Tübingen University Hospital, Tübingen, Germany; 11 Department of Neurology, University Hospital Leipzig, Leipzig, Germany; 12 Department of Neurology, Rhön-Klinikum Campus Bad Neustadt, Bad Neustadt, Germany; 13 Neurovascular Center, University Hospital Schleswig-Holstein, Campus Lübeck, Lübeck, Germany; 14 Department of Neurology, University Hospital Schleswig-Holstein, Campus Kiel, Kiel, Germany; 15 Vivantes Klinikum Neukölln, Berlin, Germany; 16 Department of Neuroradiology, Toronto Western Hospital Division of Neuroradiology, Toronto, Canada; 17 Department of Neurology, Alfried-Krupp Hospital, Essen, Germany; 18 Clinical Trial Center Würzburg, University Hospital Würzburg, Würzburg, Germany; 19 Institute for Medical Data Science, University Hospital Würzburg, Würzburg, Germany; 20 Department of Brain Sciences, Imperial College London, Room10L17, 10th Floor Lab Block, Charing Cross Campus Margravine Road, London, UK W6 8RP

**Keywords:** Oral anticoagulant, Thrombolysis, Ischemic stroke, Symptomatic intracranial hemorrhage

## Abstract

**Background:**

Approximately 10% of ischemic strokes occur in patients on oral anticoagulants (OAC), yet prospective data on hemorrhagic complications after recanalization therapies remain limited. We aimed to assess whether prior use of OAC increases the risk of intracranial hemorrhage compared to no anticoagulation in clinical routine.

**Methods:**

The prospective, multicenter RASUNOA-Prime study (clinicaltrials.gov, NCT02533960) enrolled patients with atrial fibrillation and acute ischemic stroke who were receiving a direct OAC (DOAC), a vitamin K antagonist (VKA), or no anticoagulant (non-OAC) at stroke onset. The primary endpoint was symptomatic intracranial hemorrhage (sICH) on follow-up imaging (≤ 120 h after admission), adjusted for thrombolytic therapy. Neuroimaging was centrally reviewed, blinded to treatment group.

**Results:**

Among 2,737 patients (median age 79 years, 49% female) from 46 stroke centers (DOAC n = 1,066; VKA n = 695; non-OAC n = 976), stroke severity was lower in DOAC thanin non-OAC patients (median NIHSS 4 [interquartile range (IQR) 2–9] vs. 6 [3–13]). Among those presenting within 4.5 h, thrombolysis was used less often in DOAC than in non-OAC patients (10.0% vs. 58.9%, p < 0.001), with longer door-to-needle time (+ 19 min).

The proportion of patients with any hemorrhagic transformation on admission was similar between groups. Follow-up imaging and clinical data on sICH were available for 1,813 patients (66%). Symptomatic ICH occurred in 0.59% (95% CI 0.01–1.17) of DOAC, 1.09% (95% CI 0.14–2.03) of VKA and 1.18% (95% CI 0.37–1.99) of non-OAC patients. After adjusting for thrombolysis, the odds of sICH were comparable across anticoagulation groups (adjusted OR 1.46, 95% CI 0.36–5.92, p = 0.6 for DOAC; adj. OR 1.43, 95% CI 0.43–4.70, p = 0.56 for VKA).

**Conclusions:**

The risk of sICH was not increased with DOAC use compared to no anticoagulation after ischemic stroke, with or without thrombolysis. Although event rates were low and confidence intervals wide, the findings suggest non-inferiority but cannot exclude a modest increase in bleeding risk. Randomized trials are warranted to confirm safety in this population.

**Supplementary Information:**

The online version contains supplementary material available at 10.1186/s42466-026-00462-y.

## Introduction

Approximately 10% of all patients with an ischemic stroke are taking an oral anticoagulant at the time of the event [1, 2]. This proportion is likely to increase with the growing number of patients receiving oral anticoagulation. In general, therapeutic anticoagulation is considered a contraindication for thrombolysis [3, 4], due to concerns that anticoagulants may increase the risk of cerebral hemorrhagic complications. Furthermore, the presence of anticoagulants in stroke patients who are unable to report their current medication may delay the initiation of thrombolytic therapy. This is a particular concern with direct oral anticoagulants (DOACs), for which specific coagulation assays are required to determine drug activity, potentially delaying treatment initiation. Consequently, anticoagulation represents a significant challenge for the acute management of ischemic stroke [5, 6].

Some but not all preclinical studies have found that intravenous thrombolysis is safe in anticoagulated models [7–12]. Similarly, observational data suggest that intravenous thrombolysis may be safe in patients taking DOACs [2, 13–21]. In this context, recent single-center real-world cohort data suggest that a substantial proportion of patients with reported recent DOAC intake present without relevant anticoagulant activity at admission, while symptomatic intracranial hemorrhage (sICH) rates remain low and show no clear association with measured DOAC levels or intravenous thrombolysis [22]. However, these studies were retrospective and did not include prospective enrollment or centralized, blinded analysis of brain images.

Despite these indications of potential safety, prospective data from routine clinical practice remain scarce. Therefore, the aim of our prospective study was to compare the incidence of hemorrhagic complications at admission and during the early post-stroke period in patients with atrial fibrillation (AF) who were taking a DOAC, a vitamin K antagonist (VKA), or no oral anticoagulation at stroke onset. We also analyzed the impact of thrombolysis and thrombectomy on sICH and hemorrhagic transformation.

## Methods

### Study design, standard protocol approvals, registrations, and patient consents

The Registry of Acute Stroke Under Novel Oral Anticoagulants-Prime (RASUNOA-Prime) was a prospective, multicenter, observational study of patients with AF and acute ischemic stroke or intracerebral hemorrhage (clinicaltrials.gov, identifier: NCT02533960; first posted August 27, 2015; retrospectively registered). The RASUNOA-Prime study was conducted between June 2015 and October 2019 in 46 certified stroke units in Germany and Austria. Here, we present the findings of the predefined ischemic stroke substudy. Details of the study design and the main results of the ICH substudy have been published previously [23, 24]. The study protocol was approved by the ethics committee of the Medical Faculty of the University of Heidelberg and by all local ethics committees (S-088/2015).

Adult patients with an ischemic stroke were eligible if they had known or newly diagnosed AF and stroke symptom onset within 24 h prior to admission. Patients or their legal representatives had to provide written informed consent. If a patient was unable to provide consent and died before a legal representative could be appointed, the requirement for consent was waived.

Patients were divided into three groups based on their anticoagulant treatment at the time of stroke: (1) DOAC users (apixaban, dabigatran, edoxaban, or rivaroxaban), (2) VKA users, and (3) patients without anticoagulation (non-OAC). The non-OAC group also included patients who had discontinued DOAC ≥ 72 h or VKA ≥ 7 days prior to admission. Patients with isolated retinal ischemia or those receiving therapeutic parenteral anticoagulant therapy were excluded. Where available, laboratory assessment of DOAC activity was performed at admission. Relevant anticoagulant activity was defined as DOAC plasma levels ≥ 30 ng/mL or, for dabigatran, a thrombin time (TT) ≥ 2 × the upper limit of normal (ULN), following established guidance [25].

Enrollment followed a standardized, predefined recruitment algorithm to ensure that all three treatment arms were balanced throughout the study period and to account for potential changes in stroke management over time [23]. The algorithm required that each site first enrolled a patient on a DOAC, followed by inclusion of patients into the two remaining treatment arms. The three-month survival status of each participant was determined by postal questionnaire, telephone interview with the patient or a designated representative, or by inquiry at the municipal registry office to confirm vital status.

### Neuroimaging analysis

The central adjudication of brain imaging was conducted by reviewers who were blinded to the participants´ group allocation. Each brain scan (computed tomography [CT] or magnetic resonance imaging [MRI]) was evaluated centrally using Osirix MD/Pro software (version 12.0 or later) at the core imaging laboratory by two trained, independent reviewers. In the event of a discrepancy, a third board-certified neuroradiologist was consulted. The analysis was conducted in accordance with a predefined imaging analysis manual.

All types of intracranial hemorrhages detected on admission imaging and those identified in routine follow-up scans within 120 h after admission were classified according to the Heidelberg Bleeding Classification [26]. Symptomatic ICH was defined as the occurrence of parenchymal hemorrhage (PH) type 1 or 2 combined with a worsening of the National Institutes of Health Stroke Scale (NIHSS) score by at least 4 points compared with baseline or the lowest prior NIHSS value, or death attributed to hemorrhage. In cases where clinical assessment was not possible, PH2 was considered symptomatic by definition.

Infarct volumes were determined semi-automatically using three-dimensional volumetric analysis on the latest available scan within the follow-up time period.

### Statistics

The statistical analysis was performed in accordance with the pre-specified statistical analysis plan and followed the Strengthening the Reporting of Observational Studies in Epidemiology (STROBE) guidelines for observational studies. Participant characteristics were compared using the Chi^2^ test or mid *p*-value for proportions, and the *t*-test or Mann–Whitney-*U*-test for continuous variables, depending on their distribution.

Despite the observational nature of the study, an exemplary hypothesis was formulated that the proportion of patients with sICH under DOAC therapy would be non-inferior to that of patients without oral anticoagulation. The comparison of sICH-rates between the DOAC and non-OAC, or between the VKA and non-OAC groups, was conducted using a non-inferiority test for proportions.

In accordance with a hierarchical design, the DOAC vs. non-OAC comparison was evaluated first; only if this comparison was not statistically significant, was the VKA vs. non-OAC comparison subsequently assessed.

To estimate the expected rate of sICH in stroke patients with AF without oral anticoagulation at the time of stroke, it was assumed that 15% of patients would receive intravenous thrombolysis, with a 5% sICH rate in this subgroup [27]. For the remaining 85% not receiving thrombolysis, a rate of 0.6% was assumed [28]. This resulted in an overall expected sICH rate of 1.26%.

The non-inferiority threshold was defined as a maximum doubling of the sICH rate in patients with a DOAC at the time of stroke compared with patients without anticoagulation. As no data were available at the time of study planning on sICH rates in AF patients treated with DOACs, the non-inferiority margin was directly derived from the assumed sICH rate in AF patients without OACs at stroke onset.

The planned sample size of 1,000 DOAC-treated stroke patients with AF, and 1,000 without anticoagulation ensured a power of 81% at a 5% significance level. Hence, a doubling of the 1.26% sICH rate was considered a significant deviation from the null hypothesis (inferiority). The primary endpoint was tested for non-inferiority using the Farrington–Manning score test at one-sided significance level of 2.5% with a predefined non-inferiority margin of 1.26%. Subsequently, a secondary non-inferiority test comparing VKA vs. non-OAC was performed using the same margin. To address missing follow-up imaging, we performed deterministic best- and pessimistic-case sensitivity analyses. In the best-case scenario, all missing cases were assumed not to have sICH; in the pessimistic-case scenario, early deaths (≤ 3 days) without follow-up imaging were classified as sICH. Both analyses used the same Farrington–Manning framework as the primary analysis.

To adjust for potential confounders (Alberta Stroke Program Early CT Score (ASPECTS), thrombolysis and thrombectomy), stepwise logistic regression modelling was applied. In the first step, two logistic regression models (model 1) for the occurrence of sICH adjusted for ASPECTS were calculated – one for thrombectomy and one for thrombolysis. Separately for thrombolysis and thrombectomy, the model-predicted logit of the sICH event was used as a risk score in the next step. In the second step, to compare DOAC vs. non-OAC and VKA vs. non-OAC separately, two logistic regression analyses (model 2) were performed, each adjusted for the logit-based risk score estimated in step 1.

A sensitivity analysis was conducted for secondary hemorrhagic transformation (defined as PH1/PH2). Univariable logistic regression was performed for each confounder, followed by a multivariable logistic regression model adjusted for age, NIHSS score, diabetes, infarct volume at admission, thrombectomy, and thrombolysis. Subsequently, the effects of DOAC and VKA were evaluated within the multivariable framework by adjusting for the logit of the preceding model.

## Results

### Patient and group characteristics

**Fig. 1:**
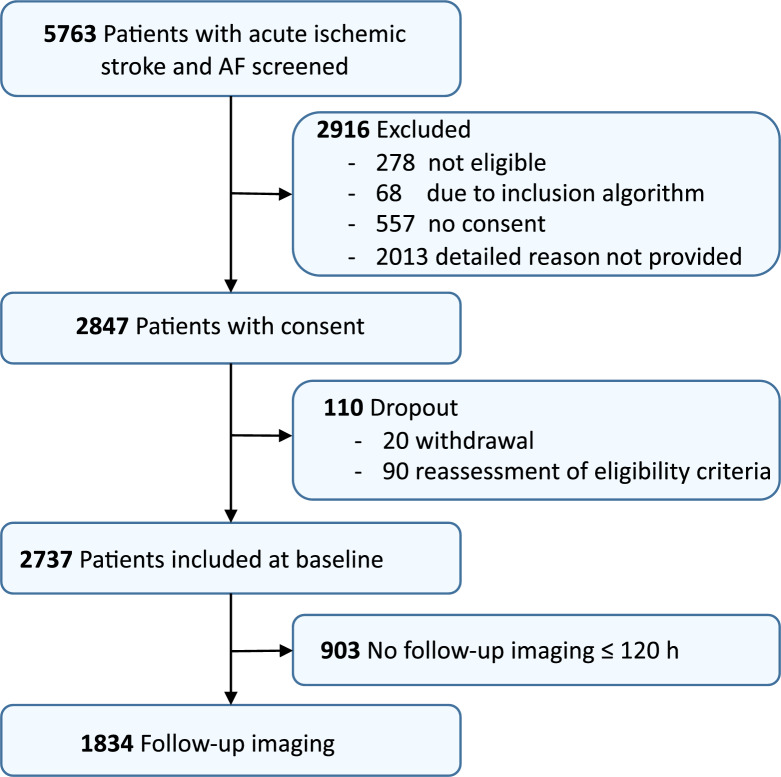
Flow chart of patient enrollment

Of 5,763 patients who underwent screening, 2,737 met the eligibility criteria. Figure 1 shows the patient flow chart. The main participant characteristics are presented in Table 1. Median age was 79 years, and 49% of participants were female. Patients who were not on oral anticoagulants were younger, had a lower prevalence of major cardiovascular risk factors, and were less likely to have a history of previous stroke/TIA or myocardial infarction compared to patients who received OACs. Additionally, patients without prior oral anticoagulation presented with more severe neurological deficits and more severe disability at admission (Table 1). Additional characteristics are shown in the Supplementary Table S1.

**Table 1:** Main characteristics of the ‘baseline cohort’ by anticoagulation scheme

**Variable**	**DOAC (N = 1066)**		**VKA (N = 695)**		**Non-OAC (N = 976)**		**p-Values**	
	**N**	**Value**	**N**	**Value**	**N**	**Value**	**DOAC vs. non-OAC**	**VKA vs. non-OAC**
Age, yr, median (IQR)	1066	79 (74–83)	695	80 (75–84)	976	78 (71–83)	0.003	< 0.001
Female sex, n (%)	1066	497 (46.6)	695	312 (44.9)	976	525 (53.8)	0.001	< 0.001
CHA_2_DS_2_-VA, median (IQR)	1061	4 (3–5)	689	4 (3–5)	973	3 (2–4)	< 0.001	< 0.001
HAS-BLED, median (IQR)	1053	2 (2–3)	686	2 (2–3)	974	2 (2–3)	< 0.001	< 0.001
Antiplatelet therapy, n (%)	1045	110 (10.5)	680	56 (8.2)	964	404 (41.9)	< 0.001	< 0.001
NIHSS at admission, median (IQR)	1055	4 (2–9)	691	5 (2–11)	973	6 (3–13)	< 0.001	0.002
modified Rankin scale score, median (IQR)								
pre-stroke	1015	0 (0–2)	667	0 (0–2)	932	0 (0–1)	< 0.001	< 0.001
at admission	1053	3 (2–4)	688	3 (2–4)	965	3 (2–4)	< 0.001	0.122
discharge	1047	2 (1–4)	689	2 (1–4)	968	2 (1–4)	0.502	0.096
Death during acute stay, n (%)	1066	53 (5)	695	36 (5.2)	976	49 (5)	0.960	0.884
Mortality at 3 months, n (%)	936	149 (15.9)	597	89 (14.9)	861	127 (14.8)	0.493	0.934
Recanalization therapy, n/N (%)								
Thrombolysis	1066	79 (7.4)	695	119 (17.1)	976	459 (47)	< 0.001	< 0.001
i.a. thrombolysis	977	3 (0.3)	639	5 (0.8)	923	31 (3.4)	< 0.001	< 0.001
Thrombectomy	1066	208 (19.5)	695	173 (24.9)	976	310 (31.8)	< 0.001	0.002

CHA_2_DS_2_-VA = Score, range 0–8, from low to high risk of ischemic stroke in atrial fibrillation; DOAC = Direct Oral Anticoagulants; HAS-BLED = HAS-BLED score, range 0–9, from low to high risk of hem orrhage under oral anticoagulation; IQR = Interquartile Range; n/N = Number of patients; NIHSS = National Institutes of Health Stroke Scale (from 0 (no symptoms) to 42)); Non-OAC = No oral anticoagulants; Rankin Scale = Modified Rankin scale score, from 0 (no symptoms) to 6 (death); VKA = Vitamin K Antagonists; i.a. thrombolysis = intra-arterial thrombolysis

A total of 1,834 patients (67%) underwent follow-up brain imaging at a median of 26.0 h (IQR 19.5–47.1) after admission. Among patients receiving any recanalization therapy, 84% (889/1058) had follow-up imaging. The baseline characteristics of patients who underwent follow-up imaging are presented in Supplementary Table S2. A comparison of patients with and without radiological follow-up is shown in Supplementary Table S3. Patients who underwent follow-up imaging were more frequently female, had a higher stroke severity at admission (median NIHSS score 6 versus 3), and had a slightly worse functional status at admission and discharge. Furthermore, patients who underwent follow-up imaging were more likely to have received thrombolysis and endovascular therapy (Table S3).

### Recanalization therapies

The proportion of patients presenting within 4.5 h after symptom onset who received intravenous thrombolysis was 31.14% (n = 633/2033). Within the 4.5 h time window, patients in the non-OAC group were more likely to be treated with thrombolysis than patients who were receiving oral anticoagulant therapy (non-OAC: 58.85% [n = 442/751]; DOAC: 10.05% [n = 76/756]; VKA: 21.86% [n = 115/526]; p < 0.001). Moreover, the median door-to-needle time was 19 min longer in patients who were taking a DOAC or a VKA than in the non-OAC group (Table S1). The proportion of patients with large-vessel occlusion who received mechanical thrombectomy did not differ between groups (Table S1).

### Evidence of any bleeding on baseline imaging

At initial presentation, any hemorrhagic transformation was observed in 85 of 2,669 (3.18%, 95% CI 2.52–3.85) patients with no statistically significant differences between the groups (Table 2).

**Table 2:** Bleeding events at baseline (all patients with evaluable imaging at admission)

	**DOAC (N = 1038)**	**VKA (N = 682)**	**Non-OAC (N = 949)**	**p-Values**	
				**DOAC vs. non-OAC**	**VKA vs. non-OAC**
**Type**				0.624	0.692
None	1010 (97.3)	658 (96.5)	916 (96.5)		
HI1	13 (1.3)	14 (2.1)	14 (1.5)		
HI2	9 (0.9)	4 (0.6)	11 (1.2)		
PH1	3 (0.3)	5 (0.7)	7 (0.7)		
PH2	2 (0.2)	1 (0.2)	1 (0.1)		
EC	1 (0.1)	0 (0.0)	0 (0.0)		

DOAC = Direct Oral Anticoagulants; Non-OAC = No oral anticoagulants; VKA = Vitamin K Antagonists. Types of hemorrhage according to the Heidelberg Bleeding Classification[26]: HI1 = Hemorrhagic infarction, Type 1 (small petechial hemorrhages, no mass effect); HI2 = Hemorrhagic infarction, Type 2 (more confluent petechial hemorrhages, no mass effect); PH1 = Parenchymal hemorrhage, Type 1 (hematoma involving ≤ 30% of the infarcted area, no substantial mass effect); PH2 = Parenchymal hemorrhage, Type 2 (hematoma involving > 30% of the infarcted area, with substantial mass effect); EC = Extracerebral hemorrhage (e.g., subarachnoid or subdural bleeding)

Data are n (%)

### Symptomatic intracranial hemorrhage in follow-up imaging according to anticoagulation status

Symptomatic ICH could be adjudicated in 1,813 of patients with follow-up imaging, and was observed in 8 of 678 patients (1.18%, 95% CI 0.37–1.99) in the non-OAC group, 4 of 675 (0.59%, 95% CI 0.01–1.17) in the DOAC and 5 of 460 (1.09%, 95% CI 0.14–2.03) in the VKA group. Based on the Farrington–Manning non-inferiority test (margin of 1.26%), sICH rates in patients taking DOACs did not exceed those in non-anticoagulated patients (crude risk difference −0.59%, p = 0.0015). Likewise, sICH rates were non-inferior for VKA compared with non-OAC (crude risk difference −0.09%). Sensitivity analyses addressing missing follow-up imaging yielded consistent results. In both the best-case scenario (all missing cases assumed event-free) and the pessimistic-case scenario (early deaths ≤ 3 days counted as sICH), non-inferiority of DOAC and VKA compared with non-OAC was maintained (Supplementary Table S4).

### Symptomatic hemorrhage following recanalization therapy

Of the 1,834 patients with follow-up imaging, 543 (29.61%) received intravenous thrombolysis with recombinant tissue plasminogen activator (rt-PA), and 589 (32.12%) underwent thrombectomy. Among patients with follow-up imaging, despite prior oral anticoagulation, 9.25% of DOAC (n = 63) and 22.27% of VKA patients (n = 104) received intravenous thrombolysis.

In univariable analysis, the odds ratio for the occurrence of a sICH was 4.44 (95% CI 1.63–12.06) for patients who received thrombolysis compared with those who did not. Taking a DOAC compared with non-OAC or a VKA compared to non-OAC did not increase the odds of sICH even after adjustment for the logit-based risk thrombolysis risk score (adjusted OR for DOAC 1.27, 95% CI 0.35–4.63, p = 0.71; adj. OR for VKA 1.42, 95% CI 0.45–4.48, p = 0.55, respectively).

Patients taking either a DOAC or a VKA did not have an increased risk of sICH compared to patients without anticoagulants (adj. OR for DOAC 0.56, 95% CI 0.17–1.89, p = 0.34; adj. OR for VKA 0.95, 95% CI 0.31–2.92, p = 0.92) after adjusting for thrombectomy.

### Factors associated with hemorrhagic transformation

The distribution of the different types of hemorrhagic transformation at follow-up imaging can be found in Table 3. The DOAC group had fewer hemorrhages in all categories than the non-OAC group.

**Table 3:** Bleeding events in follow-up imaging (all patients with evaluable follow-up imaging ≤ 120 h)

**Type**	**DOAC (N = 678)**	**VKA (N = 465)**	**Non-OAC (N = 681)**	**p-Values**	
	**Value**	**Value**	**Value**	**DOAC vs. non-OAC**	**VKA vs. non-OAC**
				0.002	0.146
None	583 (86)	354 (76.1)	535 (78.6)		
HI1	35 (5.2)	35 (7.5)	57 (8.4)		
HI2	39 (5.8)	43 (9.2)	39 (5.7)		
PH1	14 (2.1)	16 (3.4)	32 (4.7)		
PH2	6 (0.9)	13 (2.8)	16 (2.3)		
EC	1 (0.1)	4 (0.9)	2 (0.3)		

DOAC = Direct Oral Anticoagulants; Non-OAC = No oral anticoagulants; VKA = Vitamin K Antagonists. Types of hemorrhage according to the Heidelberg Bleeding Classification[26]: HI1 = Hemorrhagic infarction, Type 1 (small petechial hemorrhages, no mass effect); HI2 = Hemorrhagic infarction, Type 2 (more confluent petechial hemorrhages, no mass effect); PH1 = Parenchymal hemorrhage, Type 1 (hematoma involving ≤ 30% of the infarcted area, no substantial mass effect); PH2 = Parenchymal hemorrhage, Type 2 (hematoma involving > 30% of the infarcted area, with substantial mass effect); EC = Extracerebral hemorrhage (e.g., subarachnoid or subdural bleeding)

Data are n (%). Missings: n = 10

Factors associated with parenchymal hemorrhage in follow-up imaging were analyzed using univariable and multivariable logistic regression models (Table S5). Stroke severity according to the NIHSS score was associated with a slightly increased risk of hemorrhagic transformation (OR 1.10, 95% CI 1.07–1.12). Larger infarct volumes were associated with a substantially increased risk (> 5 to < 50 mL vs < 5 mL: OR 5.73, 95% CI 3.01–10.92; ≥ 50 mL vs < 5 mL: OR 21.73, 95% CI 11.71–40.33). Thrombolysis and thrombectomy were associated with an increased risk of secondary hemorrhagic transformation in both univariable and multivariable analyses (Table S5). Therapy with a DOAC or a VKA at the time of the stroke did not independently increase the risk of hemorrhagic transformation compared with non-OAC (Table S5).

### DOAC anticoagulation activity

In a total of 228 patients in the DOAC group DOAC concentrations or TT levels (for dabigatran) were recorded at initial presentation. Relevant anticoagulant activity was measured in 159 of the 228 patients (69.74%). Fourteen patients received thrombolysis despite the presence of relevant coagulation levels. No significant difference in the incidence of sICH was observed between patients with and without relevant laboratory-determined anticoagulant activity and follow-up imaging (sICH 1.04% [n = 1/96], 95% CI 0.00–3.07 with relevant activity versus 0% [n = 0/49] without) (Table S6).

### Clinical course and outcome

Despite baseline disparities, the functional outcome at discharge as well as in-hospital mortality and mortality at three months did not significantly differ between patients anticoagulated with DOAC or VKA and patients in the non-OAC group (Table 1).

## Discussion

The main findings of our study are that (1) sICH occurs infrequently in patients taking DOACs at the time of the stroke, and that (2) thrombolytic therapy in patients receiving DOAC therapy is not associated with a significant increase in the likelihood of symptomatic hemorrhage or any hemorrhagic transformation compared to patients without anticoagulants.

Whether oral anticoagulation increases the risk of hemorrhagic transformation—either with or without thrombolysis – is an important and unresolved issue. In large observational studies, VKA did not increase risk the of sICH after thrombolysis if the INR was ≤ 1.7 [27]. For patients who have taken a DOAC within 48 h prior to the onset of symptoms, current US and European guidelines advise against the use of IVT [3, 4]. However, preclinical studies and recent observational data on the use of DOACs prior to thrombolysis in patients challenge this recommendation [2, 13–16, 18, 19, 21]. A meta-analysis of observational studies including 3,610 patients on DOAC therapy and 243,469 without anticoagulation reported similar rates of sICH after IVT (3.4% vs. 3.5%, OR 0.95, 95% CI 0.67–1.36) [17]. Nevertheless, most included studies were retrospective, lacked centralized image review, and often did not document the exact timing of the last DOAC intake.

Our prospective study found a very low rate of sICH in stroke patients with and without oral anticoagulation. The risk of sICH after thrombolytic therapy was not significantly increased in the DOAC compared with the non-OAC group, and fulfilled the predefined non-inferiority criterion. Although patients in our study were treated with alteplase, this observation is consistent with recent real-world data comparing tenecteplase and alteplase in DOAC-treated patients, showing similarly low sICH rates for both agents [29].

While the anticoagulant effect of VKAs can be accurately ascertained through point-of-care testing [30], comparable testing for DOACs remains limited in routine clinical practice [31]. In RASUNOA-Prime, coagulation tests were available in 149 stroke patients with follow-up imaging in the DOAC group. Anticoagulation activity was relevant in 69.7%. No difference in the rate of sICH or hemorrhagic transformation was observed between patients with and without relevant laboratory determined anticoagulant activity.

A previous study even reported lower incidence of hemorrhagic transformation in stroke patients taking DOACs before thrombolysis compared with historical controls [13]. The clinical observations are supported by experimental data demonstrating that thrombin inhibition protects against secondary intracranial hemorrhage after thrombolysis [7, 8]. Thrombin – the pharmacological target of direct (dabigatran) and indirect (factor Xa-inhibitors) oral anticoagulants – can induce cytotoxic effects through activation of protease-activated receptor 1, leading to blood–brain barrier disruption, edema, and hemorrhage [18]. However, the clinical relevance of these mechanisms in human stroke remains unclear and requires further investigation.

Taken together, our findings reinforce the safety of thrombolysis in stroke patients treated with DOACs and support ongoing randomized controlled trials (e.g., DO-IT, NCT06571149, ACT-GLOBAL, NCT06352632; PASSION), which aim to confirm the safety and efficacy of IVT in this population.

It remains debated whether any hemorrhagic transformation after ischemic stroke is of clinical relevance [32–35]. Since hemorrhagic changes on admission imaging may preclude thrombolysis, understanding their true frequency across different anticoagulation regimens is essential. In our cohort, 85 of 2,669 patients (3.2%) showed hemorrhagic tranformation at admission, including 19 (0.71%) with parenchymal hemorrhage type 1 or 2 (0.48% DOAC, 0.88% VKA, and 0.84% non-OAC). Few studies have reported the incidence of hemorrhagic transformation after acute ischemic stroke in patients receiving different oral anticoagulants. In a pooled analysis of 2,183 patients from two prospective cohorts, 538 (24.6%) were on oral anticoagulation at stroke onset [36], and only 0.6% showed hemorrhagic transformation on initial imaging – a lower rate than in our study. The study also reported a lower rate of hemorrhagic transformation in follow-up imaging. This discrepancy likely reflects the longer follow-up window and the higher mean age of our cohort.

The present study has several strengths: It was prospective and enrolled patients with DOACs, VKAs, or no anticoagulation concurrently according to a predefined algorithm. Inclusion was limited to stroke patients with AF, enhancing group comparability. Centralized neuroimaging analysis was performed according to a predefined protocol by independent, blinded reviewers. However, several limitations must be acknowledged. First, group sizes were uneven, with fewer VKA patient due to declining VKA prescriptions during the study period. Second, the number of bleeding events was lower than anticipated, limiting multivariable analyses. Third, approximately 30% of patients did not undergo follow-up imaging, introducing potential bias if imaging was more likely in clinically worsened cases, overestimating hemorrhagic complications. Conversely, we cannot exclude that clinically asymptomatic bleeds were missed in patients without follow-up imaging. These patterns also limit the robustness of the non-inferiority analyses, as confirmed in best- and pessimistic-case sensitivity analyses, which —while consistent — cannot fully resolve the uncertainty introduced by selective imaging. Future studies should mandate standardized follow-up imaging protocols at fixed time points to minimize this source of bias. Fourth, stroke severity was lower in the DOAC group compared with non-OAC. Given that stroke severity was independently associated with hemorrhagic transformation, and infarct volume showed a 21-fold increased risk of hemorrhage for volumes ≥ 50 mL versus < 5 mL, this baseline imbalance represents a substantial confounder. While we attempted to adjust for this in multivariable analyses, residual confounding cannot be excluded. The lower bleeding rates in DOAC patients may partly reflect their milder initial strokes rather than the anticoagulant itself. Fifth, follow-up brain imaging was not performed at a standardized time points. Patients may have had variable DOAC absorption or adherence issues, all of which would affect bleeding risk assessment. This incomplete characterization of anticoagulant status limits our ability to assess true dose–effect relationships.

In conclusion, our prospective study indicate a low risk of intracranial bleeding in stroke patients taking a DOAC compared to no anticoagulation, both with and without thrombolysis. However, given the observational nature of this study, the small number of bleeding events, and baseline differences in stroke severity, randomized controlled trials remain essential to establish definitive safety and efficacy before clinical guidelines are revised.

## Supplementary Information

Additional file 2.

## Data Availability

Upon reasonable request to the corresponding author, anonymized data will be made available to qualified researchers for scientific purposes.

## Abbreviations

AF: Atrial fibrillation
ASPECTS: Alberta stroke program early CT score
CI: Confidence interval
CT: Computed tomography
DOAC: Direct oral anticoagulant
EC: Extracerebral hemorrhage
HI1: Hemorrhagic infarction type 1
HI2: Hemorrhagic infarction type 2
IQR: Interquartile range
INR: International normalized ratio
IVT: Intravenous thrombolysis
MRI: Magnetic resonance imaging
mRS: Modified rankin scale
NIHSS: National institutes of health stroke scale
OAC: Oral anticoagulation
OR: Odds ratio
PH1: Parenchymal hemorrhage type 1
PH2: Parenchymal hemorrhage type 2
rt-PA: Recombinant tissue plasminogen activator
sICH: Symptomatic intracranial hemorrhage
STROBE: Strengthening the reporting of observational studies in epidemiology
TT: Thrombin time
VKA: Vitamin K antagonist
