# Supplementary material for: Intracranial hemorrhage after ischemic stroke in patients on direct oral anticoagulants: results from a prospective observational study
Source: Neurol Res Pract. 2026 Feb 26;8(1):13. doi: 10.1186/s42466-026-00462-y (PMC12947503; doi:10.1186/s42466-026-00462-y)
Supplement: Supplementary file 1 — Additional file 2. [file 42466_2026_462_MOESM2_ESM.pdf]

SUPPLEMENTAL MATERIAL
RASUNOA PRIME STUDY

ONLINE SUPPLEMENTAL

Supplementary Tables

Table S1. Extended characteristics of the baseline cohort

Table S2. Characteristics of the follow-up imaging cohort

Table S3. Comparison between patients with and without follow-up imaging

Table S4. Deterministic sensitivity analyses addressing missing follow-up Imaging

Table S5. Factors associated with secondary hemorrhagic transformation

Table S6. Anticoagulation activity

Table S7. Coinvestigators and Collaborators

Supplementary Table S1. Extended characteristics of the baseline cohort

	DOAC (N=1066)		VKA (N=695)		Non-OAC (N=976)		p-Values	
**Variable**	**N**	**Value**	**N**	**Value**	**N**	**Value**	**DOAC vs. non-OAC**	**VKA vs. non-OAC**
Comorbidities, n/N (%)								
Arterial hypertension	1063	978 (92)	695	635 (91.4)	976	834 (85.5)	<0.001	<0.001
Diabetes mellitus	1055	336 (31.8)	691	260 (37.6)	973	249 (25.6)	0.002	<0.001
Hyperlipidemia	1033	508 (49.2)	677	307 (45.3)	954	371 (38.9)	<0.001	0.009
Ischemic heart disease/myocardial infarction	1055	367 (34.8)	692	236 (34.1)	971	242 (24.9)	<0.001	<0.001
Peripheral artery disease	1056	84 (8)	687	48 (7)	971	61 (6.3)	0.144	0.568
Previous Stroke/TIA	1057	276 (26.1)	693	150 (21.6)	974	111 (11.4)	<0.001	<0.001
Previous ICH	1053	14 (1.3)	690	7 (1)	973	13 (1.3)	0.990	0.553
Malignancy	1036	182 (17.6)	676	87 (12.9)	956	116 (12.1)	<0.001	0.657
Renal insufficiency	1057	132 (12.5)	692	103 (14.9)	970	93 (9.6)	0.038	0.001
Liver insufficiency	1055	14 (1.3)	691	10 (1.4)	970	18 (1.9)	0.341	0.524
Smoking	1035	120 (11.6)	676	74 (10.9)	959	118 (12.3)	0.625	0.401
Alcohol abuse	1051	43 (4.1)	689	30 (4.4)	971	64 (6.6)	0.012	0.052
Pulmonary embolism	1034	37 (3.6)	671	25 (3.7)	959	18 (1.9)	0.021	0.022
Onset to admission, minutes, median (IQR)	999	120 (58–268)	676	99 (57–244)	932	92 (52–210)	<0.001	0.105
Systolic blood pressure at admission, mean (SD)	1007	154.2 (27)	655	155.9 (27.4)	908	154.4 (28)	0.862	0.298
Glucose at admission, mg/dL, median (IQR)	993	120 (103–149)	653	121 (105–148)	909	120 (104–149)	>0.900	0.790
Type of DOAC, n/N (%)								
Apixaban	1062	429 (40.4)	.	.	.	.	.	.
Dabigatran	1062	160 (15.1)	.	.	.	.	.	.
Rivaroxaban	1062	388 (36.5)	.	.	.	.	.	.
Edoxaban	1062	85 (8)	.	.	.	.	.	.
Specific coagulation testing available, n/N (%)	1066	190 (17.8)	695	682 (98.1)	.	.	.	.
Type of atrial fibrillation, n/N (%)							<0.001	<0.001
unknown	1066	324 (30.4)	695	208 (29.9)	976	385 (39.4)		
Intermittent/paroxysmal	1066	389 (36.5)	695	180 (25.9)	976	377 (38.6)		
Permanent	1066	353 (33.1)	695	307 (44.2)	976	214 (21.9)		
ASPECTS, 6-10	680	662 (97.4)	467	449 (96.2)	684	655 (95.8)	0.107	0.746
Length-of-stay, days, median (IQR)	1065	7 (4–11)	695	8 (5-12)	975	8 (5–12)	<0.001	0.422
Referral, n/N (%)	1066	195 (18.3)	695	156 (22.4)	976	243 (24.9)	<0.001	0.247
Early palliative care, n/N (%)	1066	51 (4.8)	695	36 (5.2)	976	52 (5.3)	0.575	0.894
Discharge destination, n/N (%)							0.635	0.711
Home	1061	494 (46.6)	694	277 (39.9)	973	425 (43.7)		
Internal transfer	1061	35 (3.3)	694	23 (3.3)	973	33 (3.4)		

External transfer	1061	106 (10)	694	83 (12)	973	104 (10.7)		
Rehab	1061	334 (31.5)	694	257 (37)	973	334 (34.3)		
Nursing facility	1061	39 (3.7)	694	18 (2.6)	973	28 (2.9)		
deceased	1061	53 (5)	694	36 (5.2)	973	49 (5)		
Follow-up imaging within 120h available, n/N (%)	1066	681 (63.9)	695	467 (67.2)	976	686 (70.3)	0.002	0.178
Door-to-needle-time (thrombolysis), minutes, median (IQR)	65	54 (31–78)	100	54 (32–80)	376	35 (23–53)	<0.001	<0.001
Endovascular therapy among patients with CT-A proven large-vessel occlusion	203	103 (66.01)	167	111 (66.47)	272	188 (69.12)	0.473	0.563

Supplementary Table S2. Characteristics of the follow-up imaging cohort

	DOAC (N=681)		VKA (N=467)		Non-OAC (N=686)		p-Values	
Variable	N	Value	N	Value	N	Value	DOAC vs. non-OAC	VKA vs. non-OAC
Age, yr, median (IQR)	681	79 (74–83)	467	80 (75–84)	686	77 (70–83)	<0.001	<0.001
Female sex, n/N (%)	681	333 (48.9)	467	215 (46)	686	380 (55.4)	0.016	0.002
Comorbidities, n/N (%)								
Arterial hypertension	680	622 (91.5)	467	427 (91.4)	686	595 (86.7)	0.005	0.014
Diabetes mellitus	677	215 (31.8)	465	166 (35.7)	684	184 (26.9)	0.049	0.001
Hyperlipidemia	664	324 (48.8)	459	209 (45.5)	673	257 (38.2)	<0.001	0.014
Ischemic heart disease/myocardial infarction	678	232 (34.2)	465	160 (34.4)	684	169 (24.7)	<0.001	<0.001
Peripheral artery disease	679	56 (8.2)	463	36 (7.8)	684	43 (6.3)	0.163	0.329
Previous Stroke/TIA	678	162 (23.9)	465	95 (20.4)	686	65 (9.5)	<0.001	<0.001
Previous ICH	676	7 (1)	465	5 (1.1)	684	7 (1)	0.982	0.886
Malignancy	665	123 (18.5)	457	59 (12.9)	670	82 (12.2)	0.002	0.738
Renal insufficiency	678	89 (13.1)	465	68 (14.6)	682	64 (9.4)	0.029	0.006
Liver insufficiency	678	8 (1.2)	464	6 (1.3)	682	13 (1.9)	0.277	0.425
Smoking	663	80 (12.1)	457	52 (11.4)	680	84 (12.4)	0.873	0.620
Alcohol abuse	674	29 (4.3)	464	19 (4.1)	683	46 (6.7)	0.050	0.058
Pulmonary embolism	666	30 (4.5)	450	19 (4.2)	676	12 (1.8)	0.004	0.014
Systolic blood pressure at admission, mean (SD)	647	154.1 (27.7)	441	158 (28.2)	640	154.7 (28.6)	0.683	0.060
Glucose at admission, mg/dL, median (IQR)	637	121 (103–147)	439	120 (105–147)	642	121 (104–151)	0.489	0.462
Type of DOAC, n/N (%)								
Apixaban	679	256 (37.7)	.	.	.	.	.	.
Dabigatran	679	115 (16.9)	.	.	.	.	.	.
Rivaroxaban	679	253 (37.3)	.	.	.	.	.	.
Edoxaban	679	55 (8.1)	.	.	.	.	.	.
Specific coagulation testing available, n/N (%)	681	118 (17.3)	467	461 (98.7)	.			
Type of atrial fibrillation, n/N (%)							<0.001	<0.001
unknown	681	204 (30)	467	142 (30.4)	686	264 (38.5)		
Intermittent/paroxysmal	681	248 (36.4)	467	126 (27)	686	266 (38.8)		
Permanent	681	229 (33.6)	467	199 (42.6)	686	156 (22.7)		
CHA_2DS_2-VA, median (IQR)	681	4 (3-5)	465	4 (3-5)	686	3 (2-4)	<0.001	<0.001
HAS-BLED, median (IQR)	673	2 (2–3)	463	2 (2–3)	685	2 (2–3)	0.006	0.002

Concomitant antiplatelet therapy, n/N (%)	667	72 (10.8)	454	37 (8.1)	674	273 (40.5)	<0.001	<0.001
Onset to admission, minutes, median (IQR)	644	101 (55–241)	459	90 (53–201)	661	90 (52–190)	0.014	0.695
Referral, n/N (%)	681	137 (20.1)	467	113 (24.2)	686	169 (24.6)	0.045	0.865
NIHSS at admission, median (IQR)	673	5 (3-11)	465	7 (3-14)	683	7 (3-14)	<0.001	0.461
modified Rankin scale score, median (IQR)								
pre-stroke	648	0 (0-2)	446	0 (0-2)	654	0 (0-1)	<0.001	<0.001
at admission	673	3 (2–4)	464	4 (2–4)	680	4 (2–5)	0.008	0.923
discharge	670	3 (1–4)	466	3 (1–4)	681	2 (1–4)	0.127	0.009
Death during acute stay, n/N (%)	681	36 (5.3)	467	31 (6.6)	686	40 (5.8)	0.660	0.576
Mortality at 3 months, n/N (%)	606	97 (16)	402	73 (18.2)	606	99 (16.3)	0.876	0.451
Length-of-stay, days, median (IQR)	681	7 (4–11)	467	8 (5–13)	686	8 (5–12)	0.023	0.554
Treatment limitations (early palliative care), n/N (%)	681	34 (5)	467	31 (6.6)	686	43 (6.3)	0.306	0.801
Discharge destination, n/N (%)							0.949	0.036
Home	681	303 (44.5)	467	158 (33.8)	685	298 (43.5)		
Internal transfer	681	23 (3.4)	467	13 (2.8)	685	21 (3.1)		
External transfer	681	76 (11.2)	467	60 (12.8)	685	80 (11.7)		
Rehab	681	219 (32.2)	467	191 (40.9)	685	227 (33.1)		
Nursing facility	681	24 (3.5)	467	14 (3)	685	19 (2.8)		
deceased	681	36 (5.3)	467	31 (6.6)	685	40 (5.8)		
Recanalization therapy, n/N (%)								
i.v. Thrombolysis	681	63 (9.3)	467	104 (22.3)	686	376 (54.8)	<0.001	<0.001
i.a. thrombolysis	637	3 (0.5)	439	4 (0.9)	653	26 (4)	<0.001	0.002
Thrombectomy	681	183 (26.9)	467	150 (32.1)	686	256 (37.3)	<0.001	0.070
Door-to-needle-time (thrombolysis), minutes, median (IQR)	52	56 (37–85)	89	53 (35–82)	312	35 (23–54)	<0.001	<0.001
Infarct volume, mL, median (IQR)	655	2 (0–10)	453	2 (0.11–17.25)	665	2 (0.29–22.5)	<0.001	0.190

Supplementary Table S3. Comparison between patients with and without follow-up imaging

Variable	Total (N=2737)		w/o follow-up imaging (N = 903)		with follow-up imaging (N = 1834)		p-Value
	N	Value	N	Value	N	Value	
Age, yr, median (IQR)	2737	79 (73–83)	903	79 (74–83)	1834	79 (73–83)	0.126
Female sex, n/N (%)	2737	1334 (48.7)	903	406 (45.0)	1834	928 (50.6)	0.006
Anticoagulation schemata							0.009
None	2737	976 (35.7)	903	290 (32.1)	1834	686 (37.4)	
DOAC	2737	1066 (38.9)	903	385 (42.6)	1834	681 (37.1)	
VKA	2737	695 (25.4)	903	228 (25.2)	1834	467 (25.5)	
NIHSS at admission, median (IQR)	2719	5 (2–11)	898	3 (2–7)	1821	6 (3–13)	<0.001
modified Rankin scale score, median (IQR)							
pre-stroke	2614	0 (0–2)	866	0 (0–1)	1748	0 (0–2)	0.098
at admission	2706	3 (2-4)	889	3 (2–4)	1817	3 (2–4)	<0.001
discharge	2704	2 (1-4)	887	2 (1–3)	1817	2 (1–4)	<0.001
Treatment limitations (early palliative care), n/N (%)	2737	139 (5.1)	903	31 (3.4)	1834	108 (5.9)	0.006
Death during acute stay	2737	138 (5.0)	903	31 (3.4)	1834	107 (5.8)	0.007
Length-of-stay, days, median (IQR)	2735	8 (5–12)	901	7 (4–11)	1834	8 (5–12)	<0.001
Recanalization treatment, n/N (%)							
iv. Thrombolysis	2737	657 (24.0)	903	114 (12.6)	1834	543 (29.6)	<0.001
i.a. thrombolysis	2539	39 (1.5)	810	6 (0.7)	1729	33 (1.9)	0.026
Thrombectomy	2737	691 (25.2)	903	102 (11.3)	1834	589 (32.1)	<0.001

Supplementary Table S4. Deterministic sensitivity analyses addressing missing follow-up Imaging

Primary outcome – best case scenario (all patients with missing follow-up imaging were counted as no symptomatic ICH)

	DOAC (n=1060)	VKA (n=688)	Non-OAC (n=968)	Risk difference (DOAC vs. Non-OAC)	p-value (DOAC vs. Non-OAC) for non-inferiority*	Risk difference (VKA vs. Non-OAC)
	0.38%	0.73%	0.83%	-0.45%	<0.001	-0.10%

*non-inferiority margin: 1.26%

	Total	DOAC (n=1060)	VKA (n=688)	Non-OAC (n=968)
Symptomatic ICH, n	17	4	5	8

Primary outcome – pessimistic case scenario (deaths within 3 days counted as event for patients without follow-up imaging, all other patients without follow-up imaging were counted as no event)

	DOAC (n=1060)	VKA (n=688)	Non-OAC (n=968)	Risk difference (DOAC vs. Non-OAC)	p-value (DOAC vs. Non-OAC) for non-inferiority*	Risk difference (VKA vs. Non-OAC) (90%-CI)
Symptomatic ICH	0.85 %	1.16 %	1.14 %	-0.29%	0.0010	0.02 %

*non-inferiority margin: 1.26%

	Total	DOAC (n=1060)	VKA (n=688)	Non-OAC (n=968)
Symptomatic ICH, n	28	9	8	11

Supplementary Table S5. Factors associated with secondary hemorrhagic transformation

Model 1: Model with adjusting variables

	**univariable**	**p-value**	**multivariable**	**p-value**
			N=1744	
	**OR (95%-CI)**		**OR (95%-CI)**	
Age, years	0.990 (0.968-1.012)	0.3625	1.000 (0.973-1.027)	0.984
NIHSS, points	1.095 (1.072-1.119)	<0.0001	1.039 (1.008-1.071)	0.012
Diabetes mellitus	1.184 (0.767-1.826)	0.4461	1.231 (0.752-2.015)	0.409
Infarct volume in categories		<0.0001		<0.001
0 – 5 mL	1		1	
> 5 – < 50 mL	5.731 (3.009- 10.918)		4.905 (2.510-9.586)	
≥ 50 mL	21.730 (11.709-40.328)		14.626 (7.456-28.690)	
Thrombolysis	3.076 (2.035-4.650)	<0.0001	2.480 (1.552-3.963)	<0.001
Thrombectomy	3.901 (2.551-5.964)	<0.0001	1.795 (1.080-2.984)	0.024

Model 2: Effect of DOAC and VKA in the adjusted model

	**OR (95%-CI)**	**p-value**
**DOAC vs. non-OAC** *	0.724 (0.394-1.331)	0.298
**VKA vs. non-OAC** *	1.195 (0.699- 2.044)	0.515

* One model for DOAC vs. no anticoagulation and one model for VKA vs. no anticoagulation. Both models adjusted for the logit from the model. Outcome: severe hemorrhagic transformation (PH1 /PH2).

Supplementary Table S6. Anticoagulation activity

	Baseline (N=228)						Follow-up imaging (N=149)				
Variable	No relevant activity (N=69)		Relevant activity (N=159)		p-value		No relevant activity (N=50)		Relevant activity (N=99)		p-value
	N	Value	N	Value			N	Value	N	Value	
Thrombolysis, n/N (%)	69	11 (15.9)	159	14 (8.8)	0.113		50	9 (18)	99	14 (14.1)	0.538
Thrombectomy, n/N (%)	69	27 (39.1)	159	43 (27)	0.069		50	26 (52)	99	40 (40.4)	0.178
mRS at admission, median (IQR)	69	3 (2-5)	158	3 (2-4)	0.031		50	4 (3-5)	98	3 (2-4)	0.076
Mortality, n/N (%)											
In-hospital	69	3 (4.3)	159	9 (5.7)	0.640		50	2 (4)	99	8 (8.1)	0.400
At 3 months	61	11 (18)	142	28 (19.7)	0.780		45	7 (15.6)	89	21 (23.6)	0.280
sICH	-	-	-	-	-		49	0 (0)	96	1 (1.0)	0.669

sICH, symptomatic intracranial hemorrhage. mRS, modified Rankin scale score. Patients with available measurements at admission. Relevant activity = DOAC plasma concentration ≥ 30 ng/mL and/or thrombin time ≥ 2 ULN (dabigatran only).

Table S7. Coinvestigators and Collaborators

Name	Location	Role	Contribution
Solveig Horstmann	Heidelberg; Heidelberg University Hospital, Department of Neurology	Co-Investigator	Data acquisition
Alexandra Krauß	Heidelberg; Heidelberg University Hospital, Department of Neurology	Collaborator	Data acquisition
Caroline Renninger	Heidelberg; Heidelberg University Hospital, Department of Neurology	Collaborator	Data acquisition
Peter Ringleb	Heidelberg; Heidelberg University Hospital, Department of Neurology	Co-Investigator	Data acquisition
Ida Rangus	Berlin; Charité Berlin, Department for Neurology with experimental Neurology and Center for Stroke Research Berlin (CSB)	Co-Investigator	Data acquisition
Gerrit Maximilian Große	Hannover; Hannover Medical School	Co-Investigator	Data acquisition
Johanna Ernst	Hannover; Hannover Medical School	Co-Investigator	Data acquisition
Karin Weißenborn	Hannover; Hannover Medical School	Co-Investigator	Data acquisition
Ramona Schupper	Hannover; Hannover Medical School	Co-Investigator	Data acquisition
Hans Worthmann	Hannover; Hannover Medical School	Co-Investigator	Data acquisition
Arno Reich	Aachen; University Hospital Aachen	Co-Investigator	Data acquisition
Khouloud Poli	Tübingen; University Hospital Tübingen, Department of Neurology	Co-Investigator	Data acquisition
Johannes Tünnerhoff	Tübingen; University Hospital Tübingen, Department of Neurology	Co-Investigator	Data acquisition
Johann O. Pelz	Leipzig; University Hospital Leipzig, Department of Neurology	Co-Investigator	Data acquisition
Susanne Riebau	Lübeck; University Hospital Schleswig-Holstein, Campus Lübeck, Department of Neurology	Co-Investigator	Data acquisition
Andreas Binder	Kiel; University Hospital Schleswig-Holstein, Campus Kiel,	Co-Investigator	Data acquisition

	Department of Neurology		
Johannes Meyne	Kiel; University Hospital Schleswig-Holstein, Campus Kiel, Department of Neurology	Co-Investigator	Data acquisition
Olaf Crome	Berlin; Vivantes Klinikum Neukölln, Department of Neurology	Co-Investigator	Data acquisition
Boris Dimitrijeski	Berlin; Vivantes Klinikum Neukölln, Department of Neurology	Co-Investigator	Data acquisition
Jens Offermann	Berlin; Vivantes Klinikum Neukölln, Department of Neurology	Co-Investigator	Data acquisition
Timo Uphaus	Mainz; University Hospital Mainz, Department of Neurology	Co-Investigator	Data acquisition
Klaus Gröschel	Mainz; University Hospital Mainz, Department of Neurology	Co-Investigator	Data acquisition
Sonja Gröschel	Mainz; University Hospital Mainz, Department of Neurology	Co-Investigator	Data acquisition
Marianne Hahn	Mainz; University Hospital Mainz, Department of Neurology	Co-Investigator	Data acquisition
Elisabeth Schmid	Stuttgart; Katharinenhospital, Department of Neurology	Co-Investigator	Data acquisition
Eve Kohler	Heilbronn; SLK Kliniken Heilbronn, Department of Neurology	Co-Investigator	Data acquisition
Tobias J. Müller	Halle; Martin-Luther-University of Halle-Wittenberg, Department of Neurology	Co-Investigator	Data acquisition
Katja Wartenberg	Halle; Martin-Luther-University of Halle-Wittenberg, Department of Neurology	Co-Investigator	Data acquisition
Erendira Boss	Frankfurt; University Hospital Frankfurt a. M.	Co-Investigator	Data acquisition
Jan Hendrik Schaefer	Frankfurt; University Hospital Frankfurt a. M.	Co-Investigator	Data acquisition
Frank Hoffmann	Halle; Martha-Maria Hospital Halle	Co-Investigator	Data acquisition
Andrea Kraft	Halle; Martha-Maria Hospital Halle	Co-Investigator	Data acquisition
Bettina von Sarnowski	Greifswald; University Medicine Greifswald,	Co-Investigator	Data acquisition

	Department of Neurology		
Götz Thomalla	Hamburg; University Hospital Hamburg-Eppendorf, Hamburg	Co-Investigator	Data acquisition
Milani Deb-Chatterji	Hamburg; University Hospital Hamburg-Eppendorf, Hamburg	Co-Investigator	Data acquisition
Marc Fatar	Mannheim; University Hospital Mannheim	Co-Investigator	Data acquisition
Angelika Alonso	Mannheim; University Hospital Mannheim	Co-Investigator	Data acquisition
Michaela Wagner-Heck	Wiesbaden; Helios Dr. Horst Schmidt Kliniken Wiesbaden	Co-Investigator	Data acquisition
Frank Arne Wollenweber	Wiesbaden; Helios Dr. Horst Schmidt Kliniken Wiesbaden	Co-Investigator	Data acquisition
Peter Michels	Hamburg-Altona; Asklepios Klinik Altona, Hamburg	Co-Investigator	Data acquisition
Zoran Vukovic	Hamburg-Altona; Asklepios Klinik Altona, Hamburg	Co-Investigator	Data acquisition
Marc Pflug	Minden; Johannes Wesling Klinikum Minden	Co-Investigator	Data acquisition
Christian Urbanek	Ludwigshafen; Klinikum Ludwigshafen, Department of Neurology	Co-Investigator	Data acquisition
Sabine Schröder	Ludwigshafen; Klinikum Ludwigshafen, Department of Neurology	Co-Investigator	Data acquisition
Rainer Dziewas	Münster; University Hospital Münster	Co-Investigator	Data acquisition
Jens Minnerup	Münster; University Hospital Münster	Co-Investigator	Data acquisition
Kristian Barlinn	Dresden; University Hospital Dresden	Co-Investigator	Data acquisition
Kathrin Haase	Dresden; University Hospital Dresden	Co-Investigator	Data acquisition
Alexandra Grau	Würzburg; University Hospital Würzburg	Collaborator	Data acquisition
Udo Selig	Würzburg; University Hospital Würzburg	Collaborator	Data acquisition
Wolf-Rüdiger Schäbitz	Bielefeld; Evangelisches Krankenhaus Bielefeld	Co-Investigator	Data acquisition
Inken Piehl	Bielefeld; Evangelisches Krankenhaus Bielefeld	Co-Investigator	Data acquisition
Sebastian Jander	Düsseldorf; University Hospital Düsseldorf	Co-Investigator	Data acquisition
John-Ih Lee	Düsseldorf; University Hospital Düsseldorf	Co-Investigator	Data acquisition
Matthias Maschke	Trier, Krankenhaus der Barmherzigen Brüder	Co-Investigator	Data acquisition

Kersin Schröder	Trier, Krankenhaus der Barmherzigen Brüder	Co-Investigator	Data acquisition
Martin Nückel	Nürnberg; Klinikum Nürnberg-Süd, Paracelsis Medical Clinic, Nürnberg	Co-Investigator	Data acquisition
Frank Erbguth	Nürnberg; Klinikum Nürnberg-Süd, Paracelsis Medical Clinic, Nürnberg	Co-Investigator	Data acquisition
Albrecht Günther	Jena University Hospital	Co-Investigator	Data acquisition
Joannes Mühler	Schweinfurt; Leopoldina-Krankenhaus Schweinfurth	Co-Investigator	Data acquisition
Klaus Dötter	Schweinfurt; Leopoldina-Krankenhaus Schweinfurth	Co-Investigator	Data acquisition
Benno Ikenberg	München; Klinikum Rechts der Isar	Co-Investigator	Data acquisition
Johanna Härtl	München; Klinikum Rechts der Isar	Co-Investigator	Data acquisition
Sylke Düllberg-Boden	Herne; Evangelisches Klinikum Herne	Co-Investigator	Data acquisition
Christoph Kleinschnitz	Essen; Universitätsklinikum Essen	Co-Investigator	Data acquisition
Peter Kraft	Essen; Neurologische Universitätsklinik	Co-Investigator	Data acquisition
Pawel Kermer	Sande; Nordwest-Krankenhaus Sanderbusch	Co-Investigator	Data acquisition
Matthias Kaste	Sande; Nordwest-Krankenhaus Sanderbusch	Co-Investigator	Data acquisition
Martin Juenemann	Gießen; Universitätsklinikum Standort Gießen	Co-Investigator	Data acquisition
Tobias Braun	Gießen; Klinik und Poliklinik für Neurologie	Co-Investigator	Data acquisition
Fabian Meisel	Karlsruhe, Klinikum Karlsruhe	Co-Investigator	Data acquisition
Jürgen Hartmut Faiss	Teupitz; Asklepios Fachklinik Teupitz	Co-Investigator	Data acquisition
Uta Meyding-Lamadé	Frankfurt; Krankenhaus Nordwest	Co-Investigator	Data acquisition
Matthias W. Lorenz	Frankfurt; Neurologische Klinik	Co-Investigator	Data acquisition
Lars Marquardt	Hamburg-Wandsbek; Asklepios Klinik Wandsbek	Co-Investigator	Data acquisition
Haiko Kazarians	Hamburg-Wandsbek; Neurologie	Co-Investigator	Data acquisition

Germany, if not otherwise indicated. Local investigators and collaborators from centers who enrolled at least 1 patient in the RASUNOA-prime ischemic stroke substudy are listed.
